# Meta-Analysis of the Relative Abundance of Nuisance and Vector Mosquitoes in Urban and Blue-Green Spaces

**DOI:** 10.3390/insects13030271

**Published:** 2022-03-10

**Authors:** Charlotte G. Rhodes, Nicole A. Scavo, Micaela Finney, Juan P. Fimbres-Macias, Macey T. Lively, Brandon H. Strauss, Gabriel L. Hamer

**Affiliations:** 1 Entomology Department, Texas A&M University, College Station, TX 77843, USA; mfinney@tamu.edu (M.F.); bstrauss91@tamu.edu (B.H.S.); 2 Ecology & Evolutionary Biology Program, Texas A&M University, College Station, TX 77843, USA; 3 Veterinary Integrative Biosciences, Texas A&M University, College Station, TX 77843, USA; jfimbres@tamu.edu; 4 Epidemiology & Biostatistics Department, Texas A&M University, College Station, TX 77843, USA; maceytlively@tamu.edu

**Keywords:** mosquito, green space, blue space, urban, abundance

## Abstract

**Simple Summary:**

The increase in global urbanization has highlighted the need for sustainable infrastructure and integration of the urban and natural environment. However, there is concern that an increase in urban blue-green space may create habitats for vector species and potentially increase the risk for transmission of mosquito-borne disease. We conducted a meta-analysis to investigate the effect of urban blue-green spaces on mean mosquito abundance compared to traditional urban cities. We report that the abundance of *Aedes aegypti* is significantly higher in urban areas void of blue-green space. However, an analysis of three genera (*Aedes*, *Culex*, *Anopheles*), larval habitat guild, and specific taxa (*Aedes albopictus* and *Culex pipiens* complex) did not suggest any preference for either habitat considered. Given the lack of available studies and data focusing on contrasting mosquito abundance in blue-green spaces, we recommend that future surveillance studies quantifying mosquito abundance in urban landscapes report the quantitative data necessary to conduct meta-analyses such as this one.

**Abstract:**

Blue-green spaces (BGSs), urban areas characterized by the presence of vegetation and or water, and infrastructure form a potential solution for public health threats from increasing urbanization. We conducted a meta-analysis to test the hypothesis that blue-green spaces increase the abundance of nuisance and vector mosquito species compared to non-greened urban areas. After screening 7306 studies published since 1992, we identified 18 studies containing sufficient data from both traditional urban areas and BGSs. We found no significant difference in mean abundance of all mosquito taxa in three genera (*Aedes*, *Culex*, *Anopheles*) when comparing blue-green spaces and non-greened urban spaces. Similarly, a separate analysis of each individual genera found no significant differences. An analysis of the taxa by larval habitat guilds found no differences for container-breeding mosquitoes. Flood-water species tended to be more abundant in blue-green spaces, but the differences were not significant. The individual taxa of *Aedes albopictus* and the *Culex pipiens* complex showed no differences between blue-green and urban spaces, while the abundance of *Aedes aegypti* was significantly higher in traditional urban spaces. Due to the variety existing between and among the several types of blue-green spaces, further studies comparing each unique type of blue-green space or infrastructure will be necessary to draw conclusions regarding the influence of each structure on for urban mosquito communities.

## 1. Introduction

Cities are expanding across the globe and are predicted to house two-thirds of the world’s population by the year 2050 [1]. The rising urbanization levels pose several threats to ecological sustainability in cities, including altered nutrient flows, changes in habitat cover, alterations of disturbance regimes, shifts in species ecologies, and changes in biotic interactions [2]. Many of these changes can be mitigated using urban blue-green spaces (BGSs), defined here as land that is partially or completely covered with vegetation and that may or may not have visible water (e.g., parks, green roofs). BGSs within cities provide ecosystem services such as carbon sequestration, floodwater management, water and air purification, and habitat for wildlife [3]. Additionally, human interaction with BGSs has been shown to improve both physical and psychological health outcomes ranging from reduced obesity and diabetes to reduced cortisol levels [4]. Socially, BGSs have been linked to improved cognitive functioning, improved social networks, the facilitation of exercise, and reductions in crime and aggressive behavior [2].

Despite the multi-faceted benefits of urban BGSs, these structures often provide vegetation and water, two resources required for reproduction and development of many arthropods. As such, there is concern that certain groups of arthropods, such as mosquitoes and ticks, may increase in abundance and potentially reduce the value of urban BGSs through nuisance biting and the potential transmission of pathogens. Mosquitoes transmit disease-causing agents on a large scale, including the agents that cause malaria (vectored by *Anopheles* spp. mosquitoes) and dengue fever (vectored by *Aedes* spp. mosquitoes), resulting in an estimated 230 million [5] and 390 million [6] infections per year, respectively. Out of 112 genera of mosquitoes, there are 3 major genera involved in disease transmission among humans, namely *Aedes*, *Culex*, and *Anopheles*, which were the focus of our meta-analysis. While these genera are large and include diverse species that utilize a range of environmental habitats, they provide taxonomic groups to focus analyses on while also conducting analyses on specific species when possible.

In addition to the concerns of urban BGSs and the spread of disease, urbanization itself changes the landscape, creating new mosquito habitats and opportunities for zoonotic viral amplification and spillover events to humans [7,8]. VanAcker et al. found that the distance between urban BGSs increased both the prevalence of ticks and the risk of Lyme disease [9]. Mosquito abundance and diversity is generally higher in natural or rural areas than in urban areas [10,11,12]; however, vector species are often more prevalent in human-dominated landscapes such as cities [13,14] and are more widespread, being present in a variety of land use types [15,16]. Recent studies have shown inconclusive results when considering urban BGSs and urban space and their effects on relative mosquito abundance. Studies focusing on blue-green infrastructure show either a significantly lower [17] or a similar [18] abundance of mosquitoes when compared to nearby traditional urban infrastructure. Some studies included in our meta-analysis reported similar relative mosquito abundances between BGSs and their urban counterparts [15,19,20,21], while others show higher relative abundances in BGSs than in urban spaces [12,22]. Given these varied results, it remains unclear how urban BGS affects relative mosquito abundance.

Given that BGSs in urban ecosystems are being promoted for the health of society, it is important to consider how these features of the landscape affect mosquito populations that are a nuisance or vectors of agents of disease. The objective of this study was to synthesize published studies that report measures of mosquito abundance in BGSs and nearby urban landscapes. With these data, we test the hypothesis that urban blue-green spaces increase the relative abundance of nuisance or vector mosquito species compared to non-greened urban areas.

## 2. Materials and Methods

### 2.1. Systematic Literature Review

A systematic literature review was conducted using Web of Science and PubMed to search for peer-reviewed articles published before June 2021. Using the following search terms (mosquito * AND abundance AND urban) AND (mosquito * AND diversity AND urban) AND (mosquito * AND city) AND (mosquito * AND park), papers involving a comparison between BGSs and urban spaces were identified. The search yielded 7306 results with 658 studies left after duplicates were removed.

**Figure 1:**
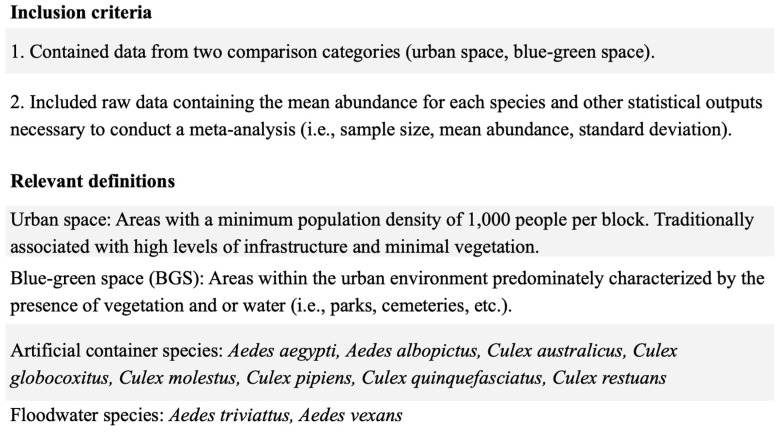
Inclusion criteria and relevant definitions.

**Figure 2:**
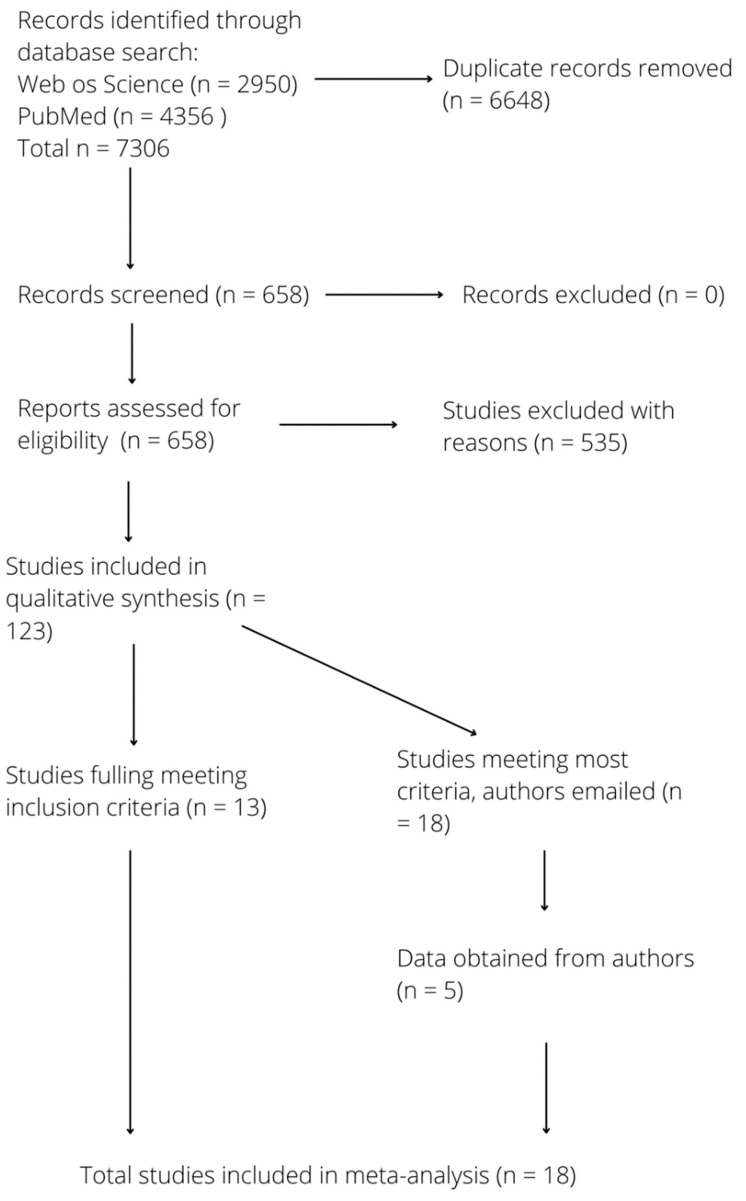
PRISMA flow diagram for systematic review and meta-analysis illustrating the number of records screened, reasons for exclusion, and number of full-text articles included in meta-analysis.

All studies were screened for relevance to the meta-analysis and scored based on the following system: (1) studies meeting all inclusion criteria (Figure 1), (2) studies meeting all the inclusion except for some of the needed components for the meta-analysis, (3) qualitatively relevant studies without the necessary data, or (4) studies that were irrelevant. Authors were emailed from category 2 to see if they were willing to share their data with us for the purposes of this study. If the authors did not respond after two emails, their studies were excluded from the analysis. The final number of studies included in the meta-analysis was 18, and 123 studies were included in the qualitative review (Figure 2).

Unrelated results were eliminated during the screening process. The abundance, mean, and standard deviation for individual mosquito species were extracted from the studies and categorized by treatment. Descriptions of the types of physical characteristics found in BGSs included in each category can be found in Table 1.

Urban spaces are typically associated with low vegetation levels and high levels of infrastructure; these areas were generally defined as urban, residential, metropolitan, or poorly vegetated suburban areas in the original papers. Blue-green spaces are highly vegetated areas within an urban environment, such as parks, cemeteries, blue-green infrastructure (BGI), wetlands, water bodies, open vegetated land, or urban farms. For a more detailed summary of the studies included in the meta-analysis, please see Supplemental Table S1.

### 2.2. Meta-Analysis

A meta-analysis was conducted to statistically compare mosquito abundance between urban and blue-green spaces. All analyses were conducted in R Version 4.0.2 following the code created by Filazzola et al. [34]. To evaluate differences in mosquito abundance in blue-green spaces versus urban spaces, the mean abundance, standard deviation, and sample size of the species abundance data were used to calculate Hedge’s g effect size [35]. Using the R package *metafor*, we fit a mixed effects model on total nuisance mosquito species (i.e., hematophagous species). A mixed effect model was used as it accounts for differences in study methodologies (e.g., trap type) [36] and sample variability that cannot otherwise be accounted for. We acknowledge that different sampling methods may attract different species and that each species has a unique ecology. Therefore, the sampling method and species were treated as moderators. A model was constructed to compare differences in abundance by species between urban spaces and blue-green spaces. To further evaluate the effect sizes within groups, we fit random effects models to three genera of medical importance—*Anopheles*, *Aedes*, and *Culex*—as well as for larval habitat guilds (artificial container breeding species and floodwater species; Figure 1) and individual taxa (*Aedes aegypti*, *Aedes albopictus*, and the *Culex pipiens* complex).

To validate the model outputs, Rosenthal’s Failsafe number was calculated for each model. Rosenthal’s Failsafe number is meant to detect bias and is expressed as the number of null results needed to invalidate the results of meta-analyses [34,37]. A higher estimate suggests high variability and that publication bias is likely not present.

## 3. Results

A total of 7306 peer-reviewed studies focusing on mosquitoes in urban areas were identified, and after duplicates were removed, 658 studies were screened. Of these, 123 studies had qualitative descriptions of mosquito species abundance in different types of spaces in or around cities, and 18 studies had quantitative data available for meta-analysis (Figure 2). All included studies were published in or after 1992, while those included in the meta-analysis were all published in or after 2001.

No significant difference in mean overall mosquito abundance between blue-green spaces and urban spaces was identified (mean effect ± SE = −0.606 ± 0.465, Z = −1.307, *p* = 0.191). However, there is a significant level of heterogeneity in the results (Q_W_ = 15,251.378, *p* < 0.001). This heterogeneity was somewhat accounted for by the mixed model (Q_E_ = 3287.259, *p* < 0.001) as we found significant differences among species and sampling method (Q_M_ = 305.696, *p* < 0.001). However, variability remains high and is most likely due to the small sample size available. Rosenthal’s Failsafe number assumes that our findings are significant and seeks to assess the presence of publication bias. Assuming our results were statistically significant, calculations of Rosenthal’s Failsafe number suggest that 21,597 unpublished studies would be needed to make the results insignificant.

Analysis of three medically important mosquito genera (i.e., *Aedes*, *Culex*, and *Anopheles*) found no significant differences in mean abundance between blue-green and urban spaces in any of the genera (Table 2). Mosquito taxa using artificial containers as larval habitats demonstrated no significant differences in abundance. This may be due to variability in BGSs sampled in each study (i.e., park vs. cemetery) and the artificial containers that may be found in those areas. Examination of floodwater species found an effect size of −2.101, suggesting a higher abundance in BGSs compared to urban spaces, but the differences were not significant. Results for the individual taxa of *Ae. albopictus* and the *Cx. pipiens* complex showed no differences between blue-green and urban spaces (*p*-value = 0.592, 0.323 respectively). Results for *Ae. aegypti* yielded a significant difference (*p* < 0.005) in mean abundance, with an effect size of 1.238, suggesting higher abundance in traditional urban areas compared to blue-green spaces. Rosenthal’s Failsafe number for *Ae. aegypti* indicates that 859 null studies are necessary to yield this observation insignificant.

## 4. Discussion

**Figure 3:**
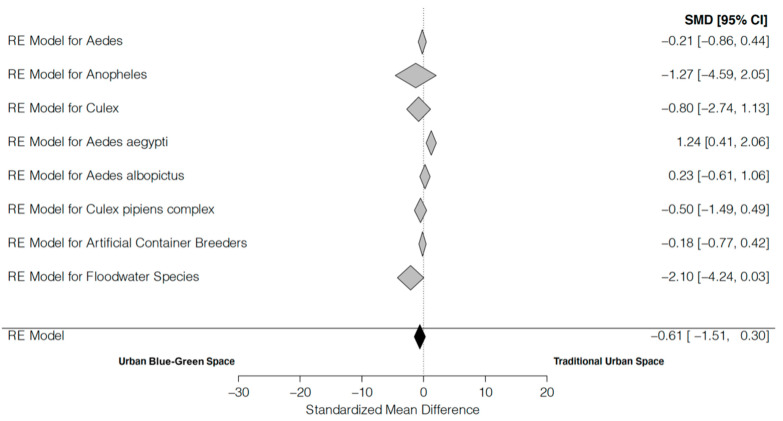
Mean effect size of blue-green space on mosquito abundance compared to traditional urban areas across multiple genera, species, and larval habitat guilds. SMD refers to the standard mean difference in abundance and the 95% confidence interval is reported in brackets.

When combining all of the mosquito species together, the mixed effects model found no difference in relative mosquito abundance between blue-green spaces versus urban spaces (Figure 3). The results of the analysis suggest that the fear of blue-green spaces increasing relative mosquito abundance overall is largely unfounded. However, our analysis did suggest that floodwater species (e.g., *Aedes vexans*) tended to be more abundant in blue-green spaces, but the difference was not significant. We did not have a sufficient number of studies to conduct the meta-analysis on each individual mosquito species, but *Ae. aegypti* abundance was significantly higher in traditional urban areas compared to BGSs. Our results are consistent with previous findings that have shown urban BGSs having a variety of effects on mosquito relative abundance [12,15,17,18,19,20,21,22], which may be based on differences in species’ ecologies and the variation present in size and structure of urban BGSs.

Species utilizing artificial containers (e.g., *Cx. pipiens* complex) tended towards having similar abundance between traditional urban spaces and urban BGSs. *Aedes aegypti*, another container-utilizing species, is known to be more prevalent in urban areas [38] and oviposit in artificial containers (e.g., water storage tanks, tires, discarded trash), which are often more prolific in anthropogenic landscapes, so our results for this species are unsurprising. This increased abundance of *Ae. aegypti*, the vector of *Aedes*-borne diseases such as dengue, Zika, and chikungunya, in traditional urban landscapes could be a mechanism supporting the observation that increasing greenness is associated with decreased human mortality [39]. Studies show that important vectors of human disease (e.g., *Culex quinquefasciatus*, *Ae. albopictus*) are present, and often dominant, across land use types (e.g., urban, BGSs) [10,13,15,16]. The conclusions from these studies support our own, as there was no significant difference in relative abundance between urban and BGSs for *Ae. albopictus* and the *Cx. pipiens* complex, suggesting that they have similar abundances in both types of space.

The lack of significant findings may be due to the variation in sampling methods, the type of blue-green spaces examined in each study, and the genera of mosquito taxa investigated. While we tried to address these issues by using a mixed model, the heterogeneity in the results was still high, indicating that a large variation was still present. A variety of BGSs exist within a city’s limits, though they vary in their biological composition, structure, and size. These differences could be explained by differences in BGSs, differing climates in different cities, and landscape connectivity and pollution [40]. For example, our BGSs criteria combined areas such as cemeteries with riparian corridors containing streams; the propensity for these two areas to flood and produce flood-water mosquitoes is likely different. Accordingly, future studies that consider additional attributes of BGSs (e.g., elevation, presence of water, and vegetation type) could yield different results. For instance, a study found that the high wind exposure on green roofs acts as a deterrent for mosquitoes [17] and the exact height of the roofs can be further addressed as a factor affecting mosquito abundance, indicating that parks and green roofs offer different microhabitats that affect mosquito abundance differently. Additionally, the biomass and composition of vegetation in BGSs influence both mosquito abundance and community composition [41], indicating that not all BGSs have the same effect on mosquito abundance. BGSs with more diverse vegetation support higher mosquito abundance [41].

While it is useful to group all BGSs together as a first step to understanding their effect on mosquito abundance, further study on their differences is warranted. However, current reporting protocols and data availability of surveillance studies make it difficult to conduct more detailed analyses at this time. This was a major challenge we faced with this meta-analysis, emphasized by the fact that we were required to reach out to authors individually for unpublished data to include in our analysis. While many studies collected surveillance data relevant to this meta-analysis, examination of relative abundance in urban BGSs or traditional urban landscapes is often not the research focus. Furthermore, many of the studies that were relevant did not report their data in a format that was usable for this analysis. Fattorini et al. [42] documented that they had similar issues with their own respective literature search in their review regarding island biogeography and urban green spaces. Many studies in urban ecology do not have the same parameters, record the same data, or report their respective analyses in the same way. Because of this, the data reported in many studies did not meet the inclusion criteria for this review. At a minimum, future surveillance studies should include in their supplemental materials the total abundance, mean, and standard deviation at the species level for each study site. Additionally, we support the system proposed by Rund et al. [43] for reporting arthropod surveillance data (i.e., MIReAD), as such a standard would eliminate many of the difficulties we faced with this meta-analysis. Conclusions for all types of green spaces increasing risk may not be suitable, and analysis for each unique type of structure, size, and maintenance schedule (among other variables) may be needed.

Another consideration for BGSs in cities and their effect on vector-borne disease transmission are the potential wildlife reservoirs in natural areas. Many of the mosquito-borne viruses present in the U.S. are zoonotic, which include wild animals as amplification reservoirs which then spill over into human populations by bridge species of mosquitoes (e.g., West Nile virus vectored by *Culex* mosquitoes). Wildlife communities in BGSs are more diverse than in traditional urban areas [34]. Furthermore, urban landscape structure may influence the distribution of reservoir hosts and thus interactions with vectors [40]. Investigation into the effects on reservoir species in the context of zoonotic vector-borne transmission warrants further consideration.

Most surveillance efforts are typically conducted during the warm and rainy months, when mosquito populations tend to increase [44]. Some of the issues encountered by this review may be explained by studies occurring during these particular periods of time. A study by Mangudo et al. found significant interaction differences between month and habitat (urban or suburban tree holes) on *Ae. aegypti* abundance between March and April [45]. With our own results, the relationship between season, abundance, and their interaction is unclear at this time and requires further research. In addition to the time of year that sampling occurred, the variation in the year each original study was conducted may have affected results. While our definition of BGSs did not change, the BGSs or urban spaces sampled in the studies could have been environments that were recently established or present for many decades which could further influence the mosquito community dynamics. Different trap types have been noted to target different mosquito species, life stages, and physiologies. However, our analysis only considers comparisons within each individual study, in which the trap types employed are consistent regardless of sampling location.

Overall, we found no significant evidence to validate the concern that BGSs will increase exposure and risk of mosquito-borne disease. While our results suggest that nuisance biting might be higher in urban BGSs than surrounding areas due to higher abundance of floodwater species, the abundance of important vector species (e.g., *Ae. aegypti*, *Cx. pipiens* complex) was not shown to be higher in BGSs than in traditional urban areas. Our results agree with other findings that suggest that BGSs are beneficial for human health and longevity [4,39]. There is still significant research needed to understand the effect of BGSs on the abundance of vector and nuisance species. Future studies should seek to validate the results suggested here and compare mosquito abundance between subcategories of BGSs.

## Figures and Tables

**Table 1:** Descriptions of blue-green space and urban spaces used for comparisons.

Study	Blue-Green Space	Urban Space
[12,19,23]	Parks and cemeteries	Suburban neighborhood
[24]	Highly vegetated suburban neighborhoods	Scarcely vegetated urban neighborhoods
[25]	Farm and park	Urban and suburban neighborhoods
[22,25]	Parks	Urban and suburban neighborhoods
[26,27,28]	Vegetated suburban areas	Scarcely vegetated urban areas
[29]	Urban forest	Peri-urban neighborhood
[30]	Urban green space	Densely urban area
[15,31]	Peri-urban neighborhood	Urban neighborhood
[10,14,20,32,33]	Suburban area	Metropolitan/urban area

**Table 2:** Standardized mean difference (SMD) of each group examined.

	SMD	Standard Error	Z Score	*p* Value
All species	−0.606	0.464	−1.307	0.191
*Aedes*	−0.211	0.333	−0.634	0.526
*Anopheles*	−1.273	1.694	−0.752	0.452
*Culex*	−0.804	0.987	−0.815	0.415
Artificial container species	−0.168	0.277	−0.608	0.543
Floodwater species	−2.102	1.090	−1.928	0.054
*Aedes aegypti*	1.238	0.421	2.944	0.003
*Aedes albopictus*	0.228	0.426	0.535	0.592
*Culex pipiens* complex	−0.498	0.504	−0.989	0.323

## Data Availability

See Supplementary Materials.

## Supplementary Materials

The following supporting information can be downloaded at: https://www.mdpi.com/article/10.3390/insects13030271/s1, A summary of the data used for meta-analysis is included in Supplemental Table S1.
