# Supplementary material for: Meta-Analysis of the Relative Abundance of Nuisance and Vector Mosquitoes in Urban and Blue-Green Spaces
Source: Insects. 2022 Mar 10;13(3):271. doi: 10.3390/insects13030271 (PMC8951424; doi:10.3390/insects13030271)
Supplement: Supplementary file 1 [file insects-13-00271-s001.zip › insects-1586619-supplementary.pdf]

Supplementary Table S1. Summary of records and data used in meta-analysis.

Study location	Year	Sampling Method	Sex	Genus	Species	Publication	Breeding	Total Abundance: BGSpace	Mean Abundance: BGSpace	Standard Deviation: BGSpace	Total Abundance: Urban	Mean Abundance: Urban	Standard Deviation: Urban
Illinois, USA	2005-2008	CDC light traps baited with CO2	Both	Total	Total	Chaves et al. 2011	NA	17024	5675	744	20471	4094	3014
Illinois, USA	2005-2008	CDC light traps baited with CO2	Both	*Aedes*	*Ae. vexans*	Chaves et al. 2011	Floodwater	4220	1406	646	3416	683	538
Illinois, USA	2005-2008	CDC light traps baited with CO2	Both	*Anopheles*	*An. punctipennis*	Chaves et al. 2011	Natural	54	18	1.7	88	17.6	13.3
Illinois, USA	2005-2008	CDC light traps baited with CO2	Both	*Culex*	*Cx. pipiens*	Chaves et al. 2011	Artificial container	9216	3072	611	14455	2911	1060
Illinois, USA	2005-2008	CDC light traps baited with CO2	Both	*Culex*	*Cx. restuans*	Chaves et al. 2011	Artificial container	487	169	27	646	192	124
Illinois, USA	2005-2008	CDC light traps baited with CO2	Both	*Aedes*	*Ae. triseriatus*	Chaves et al. 2011	Natural	1457	485	710	177	35	44
Illinois, USA	2005-2008	CDC light traps baited with CO2	Both	*Aedes*	*Ae. triviattus*	Chaves et al. 2011	Floodwater	1315	483	302	1536	271	423
Adelaide, Australia	2012-2013	CDC mini light traps baited with CO2	Both	*Culex*	*Cx. pipiens*	Johnston et al. 2014	Artificial container	461	92.2	55.37	170	34	27.61
Adelaide, Australia	2012-2013	CDC mini light traps baited with CO2	Both	*Culex*	*Cx. restuans*	Johnston et al. 2014	Artificial container	208	41.6	22.94	47	9.4	7.4
Adelaide, Australia	2012-2013	CDC mini light traps baited with CO2	Both	*Culex*	*Cx. salinarius*	Johnston et al. 2014	Saltmarsh	42	8.4	2.7	6	1.2	1.3
Adelaide, Australia	2012-2013	CDC mini light traps baited with CO2	Both	*Aedes*	*Ae. vexans*	Johnston et al. 2014	Floodwater	27	5.4	4.28	2	0.4	0.55
Adelaide, Australia	2012-2013	CDC mini light traps baited with CO2	Both	Total	Total	Johnston et al. 2014	NA	1696	565	326	2556	639	537
Adelaide, Australia	2012-2013	CDC mini light traps baited with CO2	Both	*Culex*	*Cx. quinquefasciatus*	Johnston et al. 2014	Artificial container	724	241	582	1486	421	582

Adelaide, Australia	2012-2013	CDC mini light traps baited with CO_2	Both	*Aedes*	*Ae. notoscriptus*	Johnston et al. 2014	Container - Natural and artificial	108	36	42	794	198	67
Adelaide, Australia	2012-2013	CDC mini light traps baited with CO_2	Both	*Aedes*	*Ae. vigilax*	Johnston et al. 2014	Saltmarsh	434	144	0.96	3	0.75	230
Adelaide, Australia	2012-2013	CDC mini light traps baited with CO_2	Both	*Culex*	*Cx. globocoxitus*	Johnston et al. 2014	Artificial container	362	121	111	44	11	16
Nakhan Nayok, Thailand	2008	Biogent sentinel trap, Mosquito Magnet, CDC mini light traps, aspirators	Females	*Aedes*	*Ae. aegypti*	Thongsripong et al. 2013	Artificial container	148	37	10.68	289	72.25	24.6
Nakhan Nayok, Thailand	2008	Biogent sentinel trap, Mosquito Magnet, CDC mini light traps, aspirators	Females	*Aedes*	*Ae. albopictus*	Thongsripong et al. 2013	Artificial container	13	3.25	1.5	39	9.75	5.56
Nakhan Nayok, Thailand	2008	Biogent sentinel trap, Mosquito Magnet, CDC mini light traps, aspirators	Females	*Culex*	*Cx. quinquefasciatus*	Thongsripong et al. 2013	Artificial container	1488	372	517.26	1839	459.75	372.8
Nakhan Nayok, Thailand	2008	Biogent sentinel trap, Mosquito Magnet, CDC mini light traps, aspirators	Females	*Anopheles*	An. spp.	Thongsripong et al. 2013	NA	138	34.5	11.1	227	56.75	49.94
Sao Paolo, Brazil	2001-2002	CDC mini light traps baited with CO_2	NA	Total	Total	Montes 2005	NA	742	371	742	680	340	176
Sao Paolo, Brazil	2001-2002	CDC mini light traps baited with CO_2	NA	*Aedes*	*Ae. albopictus*	Montes 2005	Artificial container	32	16	12.7	10	5	7.1
Sao Paolo, Brazil	2001-2002	CDC mini light traps baited with CO_2	NA	*Anopheles*	*An. albitarsis*	Montes 2005	Natural	4	2	2.82	1	0.5	0.7
Sao Paolo, Brazil	2001-2002	CDC mini light traps baited with CO_2	NA	*Culex*	*Cx. vaxus*	Montes 2005	NA	27	13.5	14.8	517	256	193
Rome, Italy	2012	Sticky traps	Females	*Aedes*	*Ae. albopictus*	Manica et al. 2016	Artificial container	56	5.6	1.58	60.8	7.6	0.56
Seattle, USA	2003-2004	CDC mini light traps baited with CO_2	NA	Total	Total	Pecoraro et al. 2007	NA	3287	1644	1361	1173	5894	5131
Seattle, USA	2003-2004	CDC mini light traps baited with CO_2	NA	*Culex*	*Cx. pipiens*	Pecoraro et al. 2007	Artificial container	11332	5666	4881	3282	1641	1358
Seattle, USA	2003-2004	CDC mini light traps baited with CO_2	NA	*Culex*	*Cx. tarsalis*	Pecoraro et al. 2007	Natural	3	1.5	2.12	328	164	182
Berlin, Germany	2011	Aspirators	NA	Total	Total	Honnen and Monagham 2017	NA	230	23	27.7	144	16	19.8

Berlin, Germany	2011	Aspirators	NA	*Aedes*	*Ae. vexans*	Honnen and Monagham 2017	Floodwater	25	2.5	3.2	1	0.11	0.33
Berlin, Germany	2011	Aspirators	NA	*Culex*	*Cx. pipiens*	Honnen and Monagham 2017	Artificial container	187	18.7	25.7	1136	15	19.7
Brazzaville, Republic of Congo	2017	Larval	Both	*Aedes*	*Ae. aegypti*	Wilson-Buhan et al. 2020	Artificial container	270	54	58.2	1678	229	233
Brazzaville, Republic of Congo	2017	Larval	Both	*Aedes*	*Ae. albopictus*	Wilson-Buhan et al. 2020	Artificial container	1231	246	58	438	146	153
Moreno, Pernambuco State, Brazil	2007-2008	CDC mini light traps, aspiration, ovitraps, liquid suction	NA	*Aedes*	*Ae. albopictus*	da Silva et al. 2018	Artificial container	53	10.6	14.2	365	73	92.2
Moreno, Pernambuco State, Brazil	2007-2008	CDC mini light traps, aspiration, ovitraps, liquid suction	NA	*Culex*	*Cx. quinquefasciatus*	da Silva et al. 2018	Artificial container	157	31.4	27.2	483	96.6	53.9
Adelaide, Australia	1998-2000	CDC mini light traps baited with CO2	Females	*Anopheles*	*An. annulipes*	Williams et al., 2001	Natural	793	113	126	6	3	0
Adelaide, Australia	1998-2000	CDC mini light traps baited with CO2	Females	*Culex*	*Cx. annulirostris*	Williams et al., 2001	Varies	107	15.3	22	1	0.5	0.71
Adelaide, Australia	1998-2000	CDC mini light traps baited with CO2	Females	*Culex*	*Cx. australicus*	Williams et al., 2001	Artificial container	226	32	37	12	6	1.41
Adelaide, Australia	1998-2000	CDC mini light traps baited with CO2	Females	*Culex*	*Cx. globocoxitus*	Williams et al., 2001	Artificial container	93	13.2	29.1	3	1.5	0.71
Adelaide, Australia	1998-2000	CDC mini light traps baited with CO2	Females	*Culex*	*Cx. molestus*	Williams et al., 2001	Artificial container	12	1.71	2.62	315	157.5	223
Adelaide, Australia	1998-2000	CDC mini light traps baited with CO2	Females	*Culex*	*Cx. quinquefasciatus*	Williams et al., 2001	Artificial container	3225	461	530	113	56.5	24.7
Adelaide, Australia	1998-2000	CDC mini light traps baited with CO2	Females	*Aedes*	*Ae. notoscriptus*	Williams et al., 2001	Container - Natural and artificial	1556	222	272	478	239	338
Adelaide, Australia	1998-2000	CDC mini light traps baited with CO2	Females	*Aedes*	*Ae. camptorhynchus*	Williams et al., 2001	Saltmarsh	89	12.7	21	11	5.5	6.5
Adelaide, Australia	1998-2000	CDC mini light traps baited with CO2	Females	*Aedes*	*Ae. vigilax*	Williams et al., 2001	Saltmarsh	13	1.86	4.9	1	0.5	0.71
Mercer & Monmouth counties, NJ, USA	2008	Biogent Sentinel Traps	Females	*Aedes*	*Ae. albopictus*	Unlu et al., 2011	Artificial container	7683	14.6	15.9	11269	13	15.8

Mercer & Monmouth counties, NJ, USA	2008	Biogent Sentinel Traps	Both	*Aedes*	*Ae. japonicus*	Unlu et al., 2011	Container - Natural and artificial	11	1	0	2	1	0
Mercer & Monmouth counties, NJ, USA	2008	Biogent Sentinel Traps	Both	*Aedes*	*Ae. triseriatus*	Unlu et al., 2011	Treehole	19	1	0	85	2	9.8
Mercer & Monmouth counties, NJ, USA	2008	Biogent Sentinel Traps	Both	*Aedes*	*Ae. vexans*	Unlu et al., 2011	Floodwater	46	1	0.41	11	1	0
Mercer & Monmouth counties, NJ, USA	2008	Biogent Sentinel Traps	Both	*Anopheles*	*An. quadrimaculatus*	Unlu et al., 2011	Natural	22	1	0	12	1.7	0.95
Mercer & Monmouth counties, NJ, USA	2008	Biogent Sentinel Traps	Both	*Culex*	*Cx. pipiens*	Unlu et al., 2011	Artificial container	178	2	1.8	717	1.7	9.9
Mercer & Monmouth counties, NJ, USA	2008	Biogent Sentinel Traps	Both	*Culex*	*Cx. restuans*	Unlu et al., 2011	Artificial container	128	2	1.21	24	1.14	0.35
St. Louis, MO, USA	2017-2018	Ovicups	NA	*Aedes*	*Ae. albopictus*	Westby et al, 2021	Artificial container	10108	5054	256	8712	4356	723
St. Louis, MO, USA	2017-2018	Ovicups	NA	*Aedes*	*Ae. triseriatus*	Westby et al, 2021	Treehole	27	13.5	16.2	9	4.5	3.5
St. Louis, MO, USA	2017-2018	Ovicups	NA	*Aedes*	*Ae. hendersoni*	Westby et al, 2021	Treehole	48	24	34	13	6.5	9.2
St. Louis, MO, USA	2017-2018	Ovicups	NA	*Culex*	*Cx. restuans*	Westby et al, 2021	Artificial container	616	308	436	159	79.5	112
St. Louis, MO, USA	2017-2018	Ovicups	NA	*Culex*	*Cx. pipiens*	Westby et al, 2021	Artificial container	268	134	190	651	326	260
Ghaziabad District, India	2014-2016	Hand catch	NA	*Anopheles*	*An. subpictus*	Rani et al., 2020	Natural	44	22	4.2	91	45.5	19
Tartu, Estonia	2013-2017	Hand net	Both	*Aedes*	*Ae. vexans*	Kirik et al., 2021	Floodwater	69	0.14	0.61	6	0.07	0.23
Tartu, Estonia	2013-2017	Hand net	Both	*Anopheles*	*An. claviger*	Kirik et al., 2021	Natural	3	0.006	0.1	1	0.01	0.1
Tartu, Estonia	2013-2017	Hand net	Both	*Culex*	*Cx. pipiens*	Kirik et al., 2021	Artificial container	443	0.92	2.8	96	1.1	2.5
Tartu, Estonia	2013-2017	Hand net	Both	*Culex*	*Cx. territans*	Kirik et al., 2021	Natural	38	0.08	0.4	2	0.02	0.15

Vitoria-Gasteiz, Spain	2019	Prokopac	Both	*Culex*	*Cx. pipiens*	Gonzalez et al., 2020	Artificial container	49	9.8	11.3	113	22.6	14.3
Mariana & Ouro Preto, Brazil	2011	MosquiTRAP, ovitrap	Both	*Aedes*	*Ae. aegypti*	Pedrosa et al., 2020	Artificial container	25	0.19	1.21	466	1.85	6.6
Mariana & Ouro Preto, Brazil	2011	MosquiTRAP, ovitrap	Both	*Aedes*	*Ae. albopictus*	Pedrosa et al., 2020	Artificial container	67	0.52	2.2	172	0.68	3
Tampa, FL, USA	2008	Ovitraps	NA	*Aedes*	*Ae. albopictus*	Leisnham, LaDeau, & Juliano, 2014	Artificial container	1539	10.5	21.7	402	6.81	32
Tampa, FL, USA	2008	Ovitraps	NA	*Aedes*	*Ae. aegypti*	Leisnham, LaDeau, & Juliano, 2015	Artificial container	314	2.15	6.13	269	4.5	11.8
